# T-cell and B-cell dynamics following prime-boost immunization with W135 meningococcal conjugate vaccine in mice

**DOI:** 10.3389/fimmu.2026.1900097

**Published:** 2026-08-19

**Authors:** Yahui Cheng, Guiying Kou, Haifei Zhou, Fanglei Liu, Yunjin Wang, Weiqi Wang, Aili Wu, Shuquan Luo

**Affiliations:** 1 Lanzhou Institute of Biological Products Co., Ltd., Lanzhou, China; 2 State Key Laboratory of Novel Vaccines for Emerging Infectious Diseases, China National Biotec Group Company Limited, Beijing, China

**Keywords:** B−cell activation, glycoconjugate vaccine, Neisseria meningitidis serogroup W135, T−cell activation, Th1/Th2/Th17

## Abstract

**Introduction:**

Glycoconjugate vaccines convert T-cell-independent polysaccharides into T-cell-dependent antigens, but the cellular dynamics of prime‑boost responses remain incompletely defined.

**Methods:**

Mice were immunized with the W135 meningococcal conjugate vaccine (PSW135‑TT), unconjugated PSW135, or TT alone in a two‑dose regimen given at a 2‑week interval. B‑cell and T‑cell responses were evaluated at multiple time points after each immunization.

**Results:**

PSW135‑TT accelerated PSW135‑specific IgG class switching and maintained higher IgM levels compared to PSW135 alone, with an IgG1‑dominant profile consistent with a Th2‑biased humoral response. The conjugate induced more sustained B‑cell activation across tissues and a splenic plasmablast/early plasma cell peak that coincided with antibody appearance. T‑cell analysis revealed repeated waves of early CD4⁺ T‑cell activation in blood and a triphasic late activation pattern in spleen after PSW135‑TT, suggesting prolonged activation; CD8⁺ T‑cell activation after booster was also observed. Nonspecific stimulation showed overall Th2 bias, whereas antigen‑specific restimulation induced a Th1‑dominant recall response; a late Th17 increase was observed only in the conjugate group.

**Conclusion:**

Thus, PSW135‑TT modulates B‑cell and T‑cell responses, supporting its potential as a vaccine candidate against W135 meningococcal disease.

## 1 Introduction

Neisseria meningitidis serogroup W135 remains a leading cause of invasive meningococcal disease worldwide, with recent resurgence of the highly virulent Hajj strain (MenW:cc11) linked to travel to Saudi Arabia (1, 2). In 2024–2025, travel-associated cases increased in England, and WHO confirmed infections in Umrah pilgrims (3, 4). A fatal household transmission after polysaccharide vaccination highlighted a key limitation: plain polysaccharide vaccines do not prevent nasopharyngeal carriage (5–7). Thus, more effective interventions such as glycoconjugate vaccines are urgently needed.

Capsular polysaccharide (PS) vaccines are poorly immunogenic in infants, induce short-lived immunity, and fail to generate immunological memory because they are T-cell-independent (TI) antigens (8, 9). Covalently linking PS to a carrier protein (e.g., tetanus toxoid, TT) converts it into a T-cell-dependent (TD) antigen, eliciting long-lasting protective immunity (10, 11). This “carrier effect” relies on carrier-specific CD4^+^ T cells that provide cognate help to PS-specific B cells, enabling isotype switching, affinity maturation, and memory B-cell formation (12). Mechanistically, glycoconjugates are taken up by antigen-presenting cells, processed into glycopeptides, and presented via MHC class II to activate carbohydrate-specific helper T cells (T_car_b_s_) (13).

The immune response to glycoconjugate vaccines is influenced by multiple design parameters. Several variables—including saccharide chain length, conjugation chemistry, carrier protein selection, and saccharide-to-protein ratio—can significantly affect the magnitude and quality of induced immunity (14, 15). The choice of carrier protein critically shapes B-cell and T-cell activation, and pre-exposure to a carrier can modulate anti-carbohydrate responses via either carrier priming enhancement or carrier-induced epitopic suppression (CIES) (16, 17). OMPC, a carrier protein derived from meningococcal outer membrane, exhibits intrinsic adjuvant properties through Toll-like receptor 2 (TLR2) engagement, enhancing early antibody responses and cytokine production (18, 19). The endosomal processing of glycoconjugates also plays a crucial role: in-cell depolymerization of polysaccharide antigens can modify their structure to a variable degree that correlates with the ability to activate T cells and the overall immunogenicity of the antigen (20). Clinical studies confirm that TT-based conjugate vaccines elicit robust and durable responses, with MenACWY-TT inducing significant serum bactericidal titers persisting for at least five years after primary immunization (21, 22).

The principle of glycoconjugation has been successfully applied to Haemophilus influenzae type b and pneumococcal conjugate vaccines, dramatically reducing invasive bacterial diseases (23–26). Multivalent conjugate vaccines have shown promising immunogenicity; for example, a DTaP-IPV-Hib-MCV4 vaccine in China was non-inferior to separate administrations (27), and an ACYW135 conjugate vaccine (MPCV4) in children aged 4–6 years induced higher seroconversion rates than polysaccharide vaccine (28). These findings underscore the clinical relevance of W135 conjugate vaccines.

Despite the success of licensed glycoconjugate vaccines, the detailed cellular dynamics of B-cell activation, plasmablast/early plasma cell differentiation, T-cell activation kinetics, and helper T (Th) cell polarization during primary and booster immunization remain incompletely characterized. Most studies have focused on serum antibody titers, while coordinated changes in B-cell subsets and T-cell activation markers have not been systematically analyzed at high temporal resolution. We previously reported that a conventional three-dose regimen with PSW135-TT induced stronger PSW135-specific IgG and a Th2-biased humoral response compared to plain PSW135 in mice (29). However, that study did not include antigen-specific restimulation assays, and its sampling schedule was not designed to capture early, transient events after each dose. In the present study, we adopted a simplified two-dose prime-boost regimen to focus on the dynamics of primary and booster responses, allowing direct comparison of the two immunizations and investigation of antigen-specific recall responses via restimulation.

In the present study, we performed a detailed time-course analysis of humoral and cellular immune responses in mice immunized with PSW135-TT, PSW135 alone, or TT alone under a two-dose prime-boost schedule. Our results provide a comprehensive picture of how conjugation to TT modulates the immune response, extending our previous work and supporting PSW135-TT as a vaccine candidate against W135 meningococcal disease.

## 2 Materials and methods

### 2.1 Mice

Female NIH mice (6–8 weeks old, weighing 18–22 g) were obtained from the Animal Breeding Center of Lanzhou Institute of Biological Products Co., Ltd. (Lanzhou, China; Animal Production License No.: SCXK (Gan) 2024-0001). Animals were housed under specific pathogen-free conditions at 22 ± 2 °C with a 12-h light/dark cycle and ad libitum access to food and water, acclimatized for 7 days, and randomly assigned to three groups using a computer-generated random number sequence. Sample processing and flow cytometric analysis were performed in a blinded manner. No animals were excluded from the analysis. Euthanasia was performed by CO_2_ inhalation followed by cervical dislocation. All animal experiments were approved by the Animal Care and Use Committee of the Lanzhou Institute of Biological Products Co., Ltd. (Approval No.: SYXK (Gan) 2024-0001) and carried out in accordance with institutional guidelines and the ARRIVE guidelines.

### 2.2 Antigens

PSW135 capsular polysaccharide was purified from Neisseria meningitidis serogroup W135. PSW135-TT conjugate was prepared by reductive amination. The final conjugate had a saccharide/protein ratio of 0.85 (w/w), free polysaccharide < 5%, free protein < 3%, and a molecular-size distribution (Kd = 0.45) by SEC-HPLC. Endotoxin content was < 0.1 EU/μg as determined by LAL assay. The conjugate was formulated in sterile PBS (pH 7.2) without adjuvant and stored at −20 °C until use. QC acceptance criteria: saccharide/protein ratio 0.7–1.0, free PSW135 < 5%, free protein < 3%, endotoxin < 0.1 EU/μg. Unconjugated PSW135 and TT alone were used as controls.

### 2.3 Immunization and samples collection

Female NIH mice (6–8 weeks old, weighing 18–22 g) were randomly assigned to three groups (n = 3 mice per group per time point for all assays). Each group received a subcutaneous injection (100 μL) in the inguinal region of either PSW135-TT (containing 1.25 μg of PSW135 and 1.25 μg of TT per mouse), PSW135 alone (12.5 μg/mouse), or TT alone (1.25 μg/mouse). All antigens were formulated in sterile PBS without adjuvant. A two-dose immunization schedule was used with a 14-day interval between doses (days 0 and 14).

**Figure 1:**
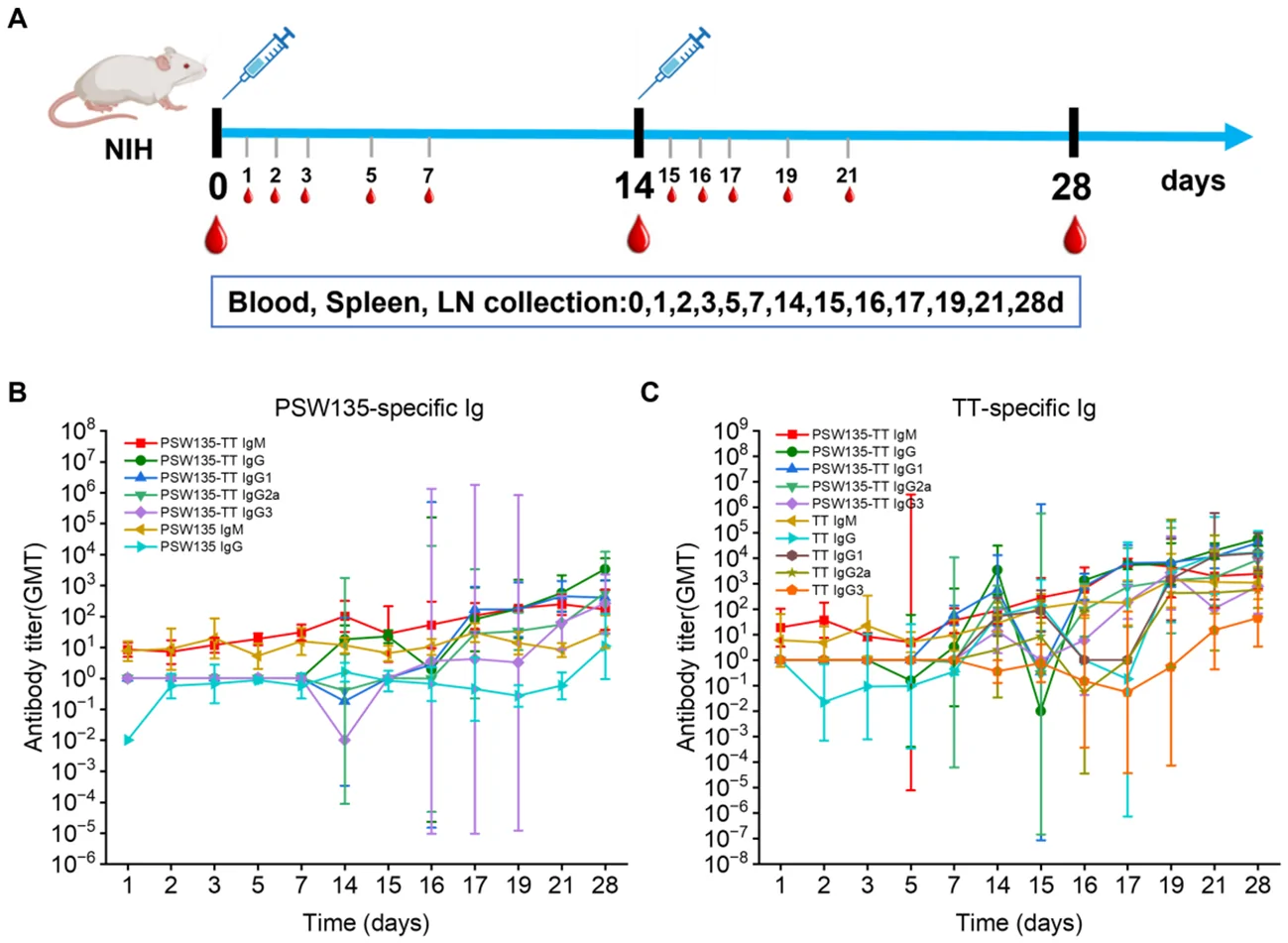
Immunization schedule and serum antibody responses. **(A)** Schematic representation of the immunization protocol. Mice received two subcutaneous injections (prime and boost) with a 14-day interval. Blood samples were collected at days 0, 1, 2, 3, 5, 7, 14, 15, 16, 17, 19, 21, and 28 (n = 3 per group per time point). **(B)** Serum levels of PSW135-specific and TT-specific antibodies (IgM, total IgG, IgG1, IgG2a, and IgG3). Data are presented as geometric mean titers (GMT) with 95% confidence intervals (error bars). Area under the curve (AUC) analysis for overall antibody responses is provided in **Supplementary Table 2**.

Blood samples (for serum separation), anticoagulated blood (for flow cytometry), draining inguinal lymph nodes, and spleens were collected from independent cohorts of mice at each time point (3 mice per group per time point) at days 0(pre-immunization), 1, 2, 3, 5, 7, 14(pre-booster), 15, 16, 17, 19, 21, and 28 (**Figure 1A**). Serum was separated by centrifugation and stored at −20 °C until analysis.

### 2.4 Cell preparation of peripheral blood, lymph nodes and spleens

Peripheral blood was collected into sodium citrate anticoagulant, and erythrocytes were removed using 1× Lysing Buffer (BD Biosciences, USA) to obtain lymphocytes. After euthanasia by CO_2_ inhalation followed by cervical dislocation, draining inguinal lymph nodes and spleens were harvested. The tissues were homogenized with glass grinders, and the resulting cell suspensions were passed through 50-μm nylon mesh to obtain single-cell suspensions. Splenic red blood cells were also lysed with Lysing Buffer (BD Biosciences, USA). The isolated lymphocytes from all three sources were washed once in RPMI 1640 medium (GIBCO, USA) supplemented with 2% fetal bovine serum (FBS, GIBCO, USA), and viable cells were enumerated using a Countess™ automated cell counter (ThermoFisher, USA) with trypan blue exclusion. Cell viability was consistently >90% for all samples.

### 2.5 Serum antigen-specific immunoglobulin titers

PSW135- and TT-specific antibody titers in serum were determined by standardized ELISA. Briefly, 96-well plates were coated with PSW135 polysaccharide (2 μg/mL) or TT (1 μg/mL) in carbonate-bicarbonate buffer (pH 9.6) overnight at 4 °C. After blocking with 1% BSA in PBST for 2 h at 37 °C, serial two-fold dilutions of test sera and reference standard serum were added and incubated overnight at 4 °C. Alkaline phosphatase-conjugated goat anti-mouse IgM, total IgG, IgG1, IgG2a, or IgG3 (Southern Biotech, USA) were then added, followed by PNPP substrate (Sigma-Aldrich, USA). Absorbance was measured at 405 nm. Antibody titers were calculated using a four-parameter logistic standard curve. The limit of detection (LOD) was defined as the lowest dilution with an absorbance exceeding the mean + 3 SD of blank wells. Samples with undetectable titers were assigned a value of 1 (1:1) for log-transformation, as this value was below the lowest observed titer in the assay. Intra-assay and inter-assay coefficients of variation were < 10% and < 15%, respectively. Results are expressed as geometric mean titers (GMT) with 95% confidence intervals (CIs).

### 2.6 Lymphocyte surface markers and flow cytometric analysis

Single-cell suspensions from blood, draining inguinal lymph nodes, and spleen were stained with fluorochrome-conjugated anti-mouse antibodies after Fc block (anti-CD16/CD32). The following antibodies were used (all from BD Biosciences): CD3-PerCP-Cy5.5, CD4-FITC, CD8-PE, CD69-APC, CD25-PE, CD19-PE, and CD138-APC. At least 50,000 lymphocyte-gated events were acquired on a BD FACScalibur cytometer. Compensation was performed with single-stained cells, and FMO controls were used for all markers. Doublet exclusion was based on FSC-A vs FSC-H, and lymphocytes were gated on FSC-A vs SSC-A. Gating hierarchy: lymphocytes → CD3^+^ (T cells)/CD19^+^ (B cells); CD4^+^ and CD8^+^ subsets; CD69^+^ and CD25^+^ activation markers; CD19^+^CD138^+^ (plasmablast/plasma cells). Representative gating strategies for all tissues are shown in **Supplementary Figure 1**. Data were analyzed with FlowJo.

### 2.7 Intracellular cytokine staining and flow cytometric analysis

For intracellular cytokine staining, single-cell suspensions from blood, draining inguinal lymph nodes, and spleen were cultured in RPMI 1640 medium supplemented with 10% FBS, penicillin, and streptomycin.

For non-specific stimulation, cells were stimulated with PMA (50 ng/mL) plus ionomycin (1 μg/mL) for 1 h at 37 °C, followed by the addition of Brefeldin A (10 μg/mL, BD Biosciences) for an additional 4 h.

For antigen-specific stimulation, cells were stimulated with PSW135-TT (5 μg/mL) or TT (2.5 μg/mL) for 72 h at 37 °C in 5% CO_2_. Brefeldin A (10 μg/mL) was added for the final 4 h of culture.

After stimulation, cells were washed twice with FACS buffer (PBS containing 2% FBS), then surface-stained with CD4-FITC for 30 min at 4 °C in the dark. Following washing, cells were fixed and permeabilized using BD Fixation/Permeabilization Solution for 20 min at 4 °C and washed with 1× BD Perm/Wash buffer. Intracellular staining was performed with IFN-γ-PerCP-Cy5.5, IL-17A-PE, and IL-4-APC (all from BD Biosciences) for 1 h at 4 °C in the dark. After washing, cells were acquired on a BD FACScalibur flow cytometer, and data were analyzed with FlowJo software. Th1, Th2, and Th17 cells were defined as CD4^+^IFN-γ^+^, CD4^+^IL-4^+^, and CD4^+^IL-17A^+^, respectively.

### 2.8 Statistical analysis

All statistical analyses and graphing were performed using OriginPro 2023b. Antibody titers were natural log-transformed prior to analysis, and cell frequencies (%) were logit-transformed [ln(p/(1-p))] to satisfy normality assumptions. For time-course data, each tissue was analyzed separately using two-way ANOVA with treatment group and time as fixed factors, including their interaction term (Group × Time). Tukey’s HSD post-hoc test was used for pairwise comparisons, and the Benjamini–Hochberg false discovery rate (FDR) procedure was applied to control for multiple comparisons; adjusted p-values (p_adj) < 0.05 were considered significant. Effect sizes are reported as partial eta squared (η²p), with 0.01, 0.06, and 0.14 interpreted as small, medium, and large effects, respectively. Area under the curve (AUC) was calculated from GMT kinetics using the trapezoidal rule with baseline Y = 0 to quantify overall antibody responses. Data are presented as estimated marginal means with 95% confidence intervals (CIs).

## 3 Results

### 3.1 Kinetics of PSW135-specific and TT-specific antibody levels

Serum levels of PSW135-specific and TT-specific antibodies were measured by ELISA following immunization (n = 3 mice per group per time point). For anti-PSW135 antibodies, IgM was detectable in all groups but declined markedly by day 28 after the booster immunization. Anti-PSW135 IgG was first detected on day 14 in the PSW135-TT group (GMT = 17.95, 95% CI: 6.24–51.63), whereas the PSW135 group showed only weak levels by day 28 (GMT = 10.88, 95% CI: 0.95–124.79) (**Figure 1B**). The PSW135-TT group exhibited a continuous upward trend in PSW135-specific antibodies after the booster immunization, while the PSW135 group showed relatively stable low levels throughout the observation period.

For anti-TT antibodies, IgG and IgG1 were detectable as early as day 7 post-immunization in both the PSW135-TT and TT groups (**Figure 1C**). IgG subclass analysis revealed that IgG1 levels were higher than IgG2a and IgG3 for both PSW135-specific and TT-specific antibodies across all groups.

Throughout the immunization period, both PSW135-specific and TT-specific IgM levels were higher in the PSW135-TT group than in the PSW135 or TT alone groups (**Figures 1B, C**). Quantitative analysis by AUC confirmed that the PSW135-TT group induced the highest overall antibody responses for both antigens (**Supplementary Table 2**).

### 3.2 Kinetics of B cell subsets in the blood, lymph nodes and spleen: total B cells, activated B cells and plasma cells

The frequencies of total B cells (CD19^+^), activated B cells (CD19^+^CD40^+^), and plasma cells (CD19^+^CD138^+^) in the blood, lymph nodes (LN), and spleen were measured by flow cytometry (n = 3 per group per time point). Two-way ANOVA revealed significant Group × Time interactions for activated B cells in the spleen (F = 0.92, p = 0.576,η²p = 0.221)and plasma cells in the spleen (F = 13.47, p < 0.001,η²p = 0.806) (**Supplementary Table 3A**).

**Figure 2:**
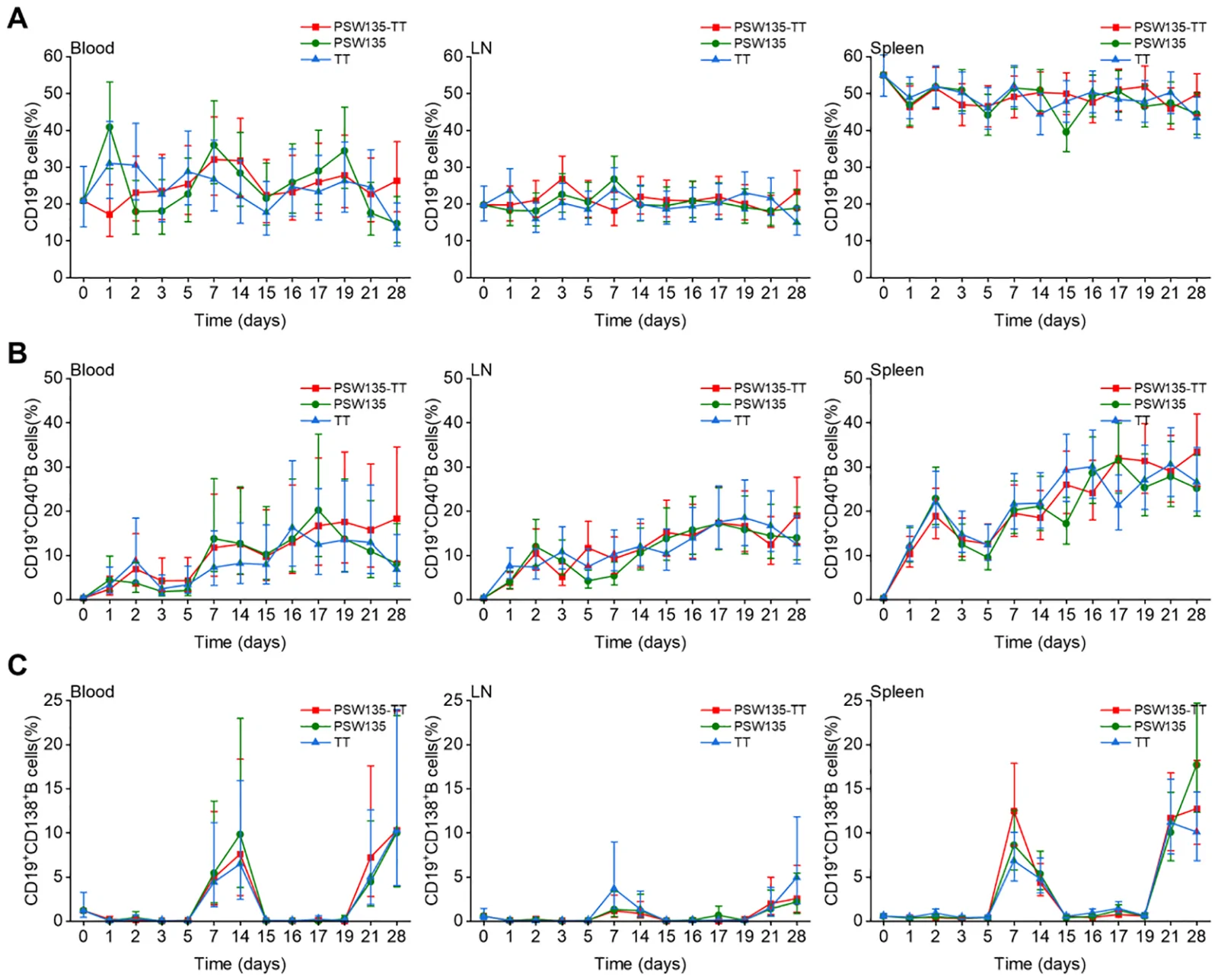
Kinetics of B cell subsets in blood, lymph nodes (LN), and spleen. Mice (n = 3 per group per time point) received two subcutaneous injections (prime at day 0, boost at day 14) of PSW135-TT, PSW135 alone, or TT alone. Flow cytometric analysis was performed at days 0, 1, 2, 3, 5, 7, 14, 15, 16, 17, 19, 21, and 28. Frequencies of **(A)** total B cells (CD19^+^), **(B)** activated B cells (CD19^+^CD40^+^), and **(C)** plasma cells (CD19^+^CD138^+^) in blood, LN, and spleen. Data are presented as estimated marginal means with 95% confidence intervals (error bars). Statistical analysis was performed as described in Section 2.8; full ANOVA results are provided in **Supplementary Table 3A**.

Across all tissues, B cell frequencies were higher in the spleen than in blood and LN. Activated B cells showed an overall upward trend in all groups, while plasma cells were detected at low levels during the first 5 days after primary immunization, followed by a marked increase by day 7 (**Figures 2A–C**).

In the blood, total B cells in the PSW135 group peaked on day 1, earlier than the TT group (day 2) and the PSW135-TT group (day 7). After booster immunization, total B cells in the PSW135 and TT groups declined to their lowest levels by day 28, whereas the PSW135-TT group remained relatively stable (**Figure 2A**). Activated B cells increased sharply on day 2 and decreased by day 5. In the PSW135 and TT groups, activated B cells declined again after booster immunization (days 17–19), whereas the PSW135-TT group showed a sustained upward trend, most notably in the spleen (**Figure 2B**). Plasma cells peaked in the spleen on day 7, with the highest levels in the PSW135-TT group. In blood, plasma cells peaked on day 14 and then fell below the detection limit. After booster immunization, plasma cell levels increased again from day 19, with the PSW135 group reaching the highest splenic levels on day 28 (**Figure 2C**).

### 3.3 Kinetics of total T cells, CD3^+^CD4^+^ T cells and CD3^+^CD8^+^ T cells frequencies in blood, lymph nodes and spleens

The frequencies of total T cells (CD3^+^), CD4^+^ T cells (CD3^+^CD4^+^), and CD8^+^ T cells (CD3^+^CD8^+^) in the blood, lymph nodes (LN), and spleen of immunized mice were measured by flow cytometry (n = 3 per group per time point). Two-way ANOVA revealed significant Group × Time interactions for CD8^+^ T cells in LN (F = 1.61, p = 0.061, η²p = 0.331) (**Supplementary Table 3B**).

**Figure 3:**
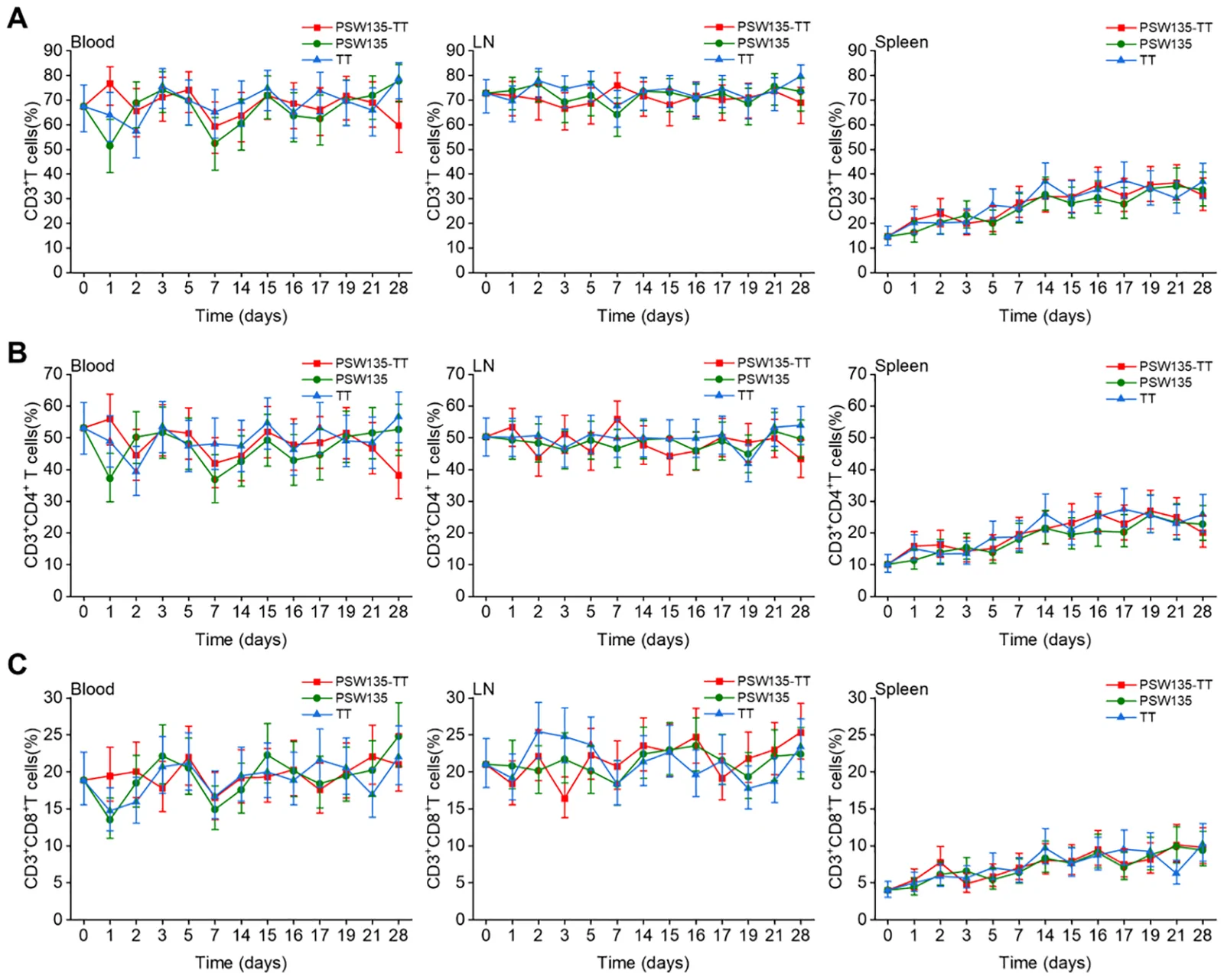
Kinetics of T cell subsets in blood, lymph nodes (LN), and spleen. Mice (n = 3 per group per time point) received two subcutaneous injections (prime at day 0, boost at day 14) of PSW135-TT, PSW135 alone, or TT alone. Flow cytometric analysis was performed at days 0, 1, 2, 3, 5, 7, 14, 15, 16, 17, 19, 21, and 28. Frequencies of **(A)** total T cells (CD3^+^), **(B)** CD4^+^ T cells, and **(C)** CD8^+^ T cells in blood, LN, and spleen. Data are presented as estimated marginal means with 95% confidence intervals (error bars). Statistical analysis was performed as described in Section 2.8; full ANOVA results are provided in **Supplementary Table 3B**.

Overall, the proportions of all T cell populations were higher in the blood and LN than in the spleen, and the percentage of CD4^+^ T cells was higher than that of CD8^+^ T cells across all tissues (**Figures 3A–C**). In the LN, the proportions of total T cells and their subsets remained relatively stable across all three groups. In contrast, total T cells, CD4^+^ T cells, and CD8^+^ T cells in the spleen all showed a gradual upward trend over time (**Figures 3A–C**).

In the blood, total T cells and CD4^+^ T cells fluctuated markedly. In the PSW135 group, both populations dropped to their lowest levels on days 1 and 7. In the TT group, they showed a transient decrease on day 2 and remained around 70% thereafter. In the PSW135-TT group, they reached a nadir on day 7, subsequently stabilized at approximately 70%, and declined again on day 28 (**Figures 3A, B**).

CD8^+^ T cells displayed distinct dynamics. In the blood, the kinetic profile of CD8^+^ T cells was generally similar to that of total and CD4^+^ T cells. However, in the LN, the TT group showed a peak on day 2; the PSW135-TT group dropped to its lowest level on day 3 and then increased markedly from day 17 onward; no obvious fluctuations were observed in the PSW135 group (**Figure 3C**). In the spleen, CD8^+^ T cells remained relatively stable, consistent with the trends of total and CD4^+^ T cells.

In the blood, total T cells and CD4^+^ T cells in the PSW135 group exhibited two troughs with relatively low levels, whereas those in the PSW135-TT group showed overall minor fluctuations, with only transient declines on days 7 and 28. In the LN, CD8^+^ T cells in the PSW135-TT group showed a late-stage increase, while those in the TT group showed an early peak (**Figures 3A–C**).

### 3.4 Kinetics of activated CD3^+^CD4^+^ and CD3^+^CD8^+^ T cells frequencies in blood, lymph nodes and spleens

**Figure 4:**
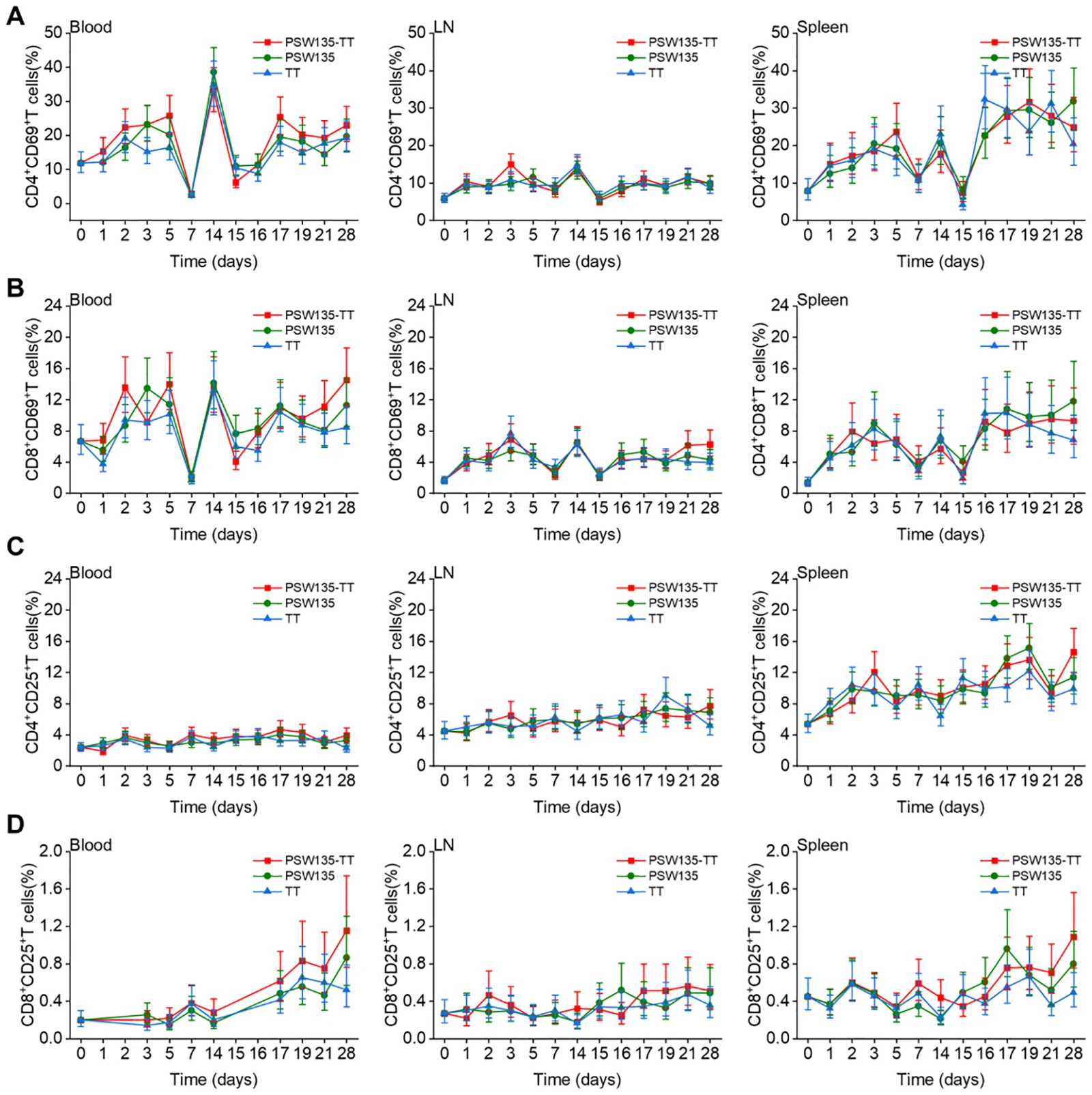
Kinetics of activated T cell subsets in blood, lymph nodes (LN), and spleen. Mice (n = 3 per group per time point) received two subcutaneous injections (prime at day 0, boost at day 14) of PSW135-TT, PSW135 alone, or TT alone. Flow cytometric analysis was performed at days 0, 1, 2, 3, 5, 7, 14, 15, 16, 17, 19, 21, and 28. Frequencies of **(A)** activated CD4^+^ T cells (CD4^+^CD69^+^), **(B)** activated CD8^+^ T cells (CD8^+^CD69^+^), **(C)** CD4^+^CD25^+^ T cells, and **(D)** CD8^+^CD25^+^ T cells in blood, LN, and spleen. Data are presented as estimated marginal means with 95% confidence intervals (error bars). Statistical analysis was performed as described in Section 2.8; full ANOVA results are provided in **Supplementary Table 3C**.

The frequencies of early activation marker CD69^+^ and late activation marker CD25^+^ on CD4^+^ and CD8^+^ T cells were assessed in the blood, lymph nodes (LN), and spleen (n = 3 per group per time point). Two-way ANOVA revealed significant Group × Time interactions for activated CD4^+^ T cells in the blood (F = 1.61, p = 0.061, η²p = 0.331) and activated CD8^+^ T cells in the blood (F = 1.56, p = 0.073, η²p = 0.324) (**Supplementary Table 3C**). Activation characteristics varied among tissues: dynamic changes in the blood and spleen showed multiple fluctuations, whereas changes in the LN were relatively stable. CD4^+^ T cells consistently showed higher activation levels than CD8^+^ T cells across all tissues (**Figure 4**).

Early activation (CD69^+^): In the blood, CD4^+^CD69^+^ T cells in the PSW135-TT group showed slightly higher proportions compared to the other two groups on days 1–5 after the first immunization and days 16–28 after the second immunization (**Figure 4A**). These cells exhibited multiple fluctuations: they increased from day 1 to day 5, decreased to a nadir on day 7, then increased again and peaked on day 14. After the second immunization, they decreased on day 15, increased on day 17, slowly declined, and rose again on day 21 (**Figure 4A**). In the PSW135 group, CD4^+^CD69^+^ T cells first peaked on day 3, with subsequent trends similar to those in the PSW135-TT group. In the TT group, they first peaked on day 2, with a generally comparable pattern thereafter (**Figure 4A**). In the LN, CD4^+^CD69^+^ T cells were fewer in number; they first peaked on day 3 in the PSW135-TT group, and all three groups showed a second peak on day 14, with largely consistent trends (**Figure 4A**). In the spleen, the dynamics were similar to those in the blood but showed an overall upward trend. In the PSW135-TT group, they peaked on day 5, decreased on day 7, and after the second immunization, they peaked again on day 19. This second peak occurred later than in the TT group (day 16) and the PSW135 group (day 17) (**Figure 4A**). On days 16–17, the TT group showed higher levels than the other two groups. CD8^+^CD69^+^ T cells showed similar kinetic patterns to their CD4^+^ counterparts but were present at lower frequencies overall (**Figure 4B**).

Late activation (CD25^+^): In the blood, CD4^+^CD25^+^ T cells showed no obvious fluctuations in any of the three groups. In the LN, these cells exhibited a slow upward trend with modest changes overall, and only the TT group showed a single peak on day 19 (**Figure 4C**). In the spleen, CD4^+^CD25^+^ T cells showed an overall upward trend. In the PSW135-TT group, they first peaked on day 3 at levels higher than those in the other two groups, then slowly increased and reached a second peak on day 19 (lower than that in the TT group), and subsequently rose again to the highest level on day 28, remaining higher than those in the other two groups (**Figure 4C**). In contrast, both the PSW135 and TT groups peaked only once, on day 19 (**Figure 4C**). CD8^+^CD25^+^ T cells were present at the lowest frequencies overall. In the blood, these cells increased after the second immunization in all three groups, with the most pronounced increase in the PSW135-TT group (**Figure 4D**). In the spleen, these cells also increased most significantly in the PSW135-TT group after the second immunization, whereas no notable changes were observed in the LN (**Figure 4D**).

### 3.5 Th1, Th2, and Th17 cell responses

#### 3.5.1 Th cell subsets induced by non-specific stimulation

To explore changes in Th cell subsets induced by the three different immunogens, peripheral blood mononuclear cells, lymph node cells, and splenocytes were stimulated non-specifically with PMA and ionomycin. The proportions of Th1 (CD4^+^IFN-γ^+^), Th2 (CD4^+^IL-4^+^), and Th17 (CD4^+^IL-17A^+^) cells were detected by intracellular cytokine staining (n = 3 per group per time point). Two-way ANOVA revealed significant Group × Time interactions for Th1, Th2, and Th17 cells across multiple tissues (**Supplementary Table 4A**). Overall, Th2 cells were consistently higher than Th1 and Th17 cells across most tissues and time points, indicating a Th2-biased response pattern.

**Figure 5:**
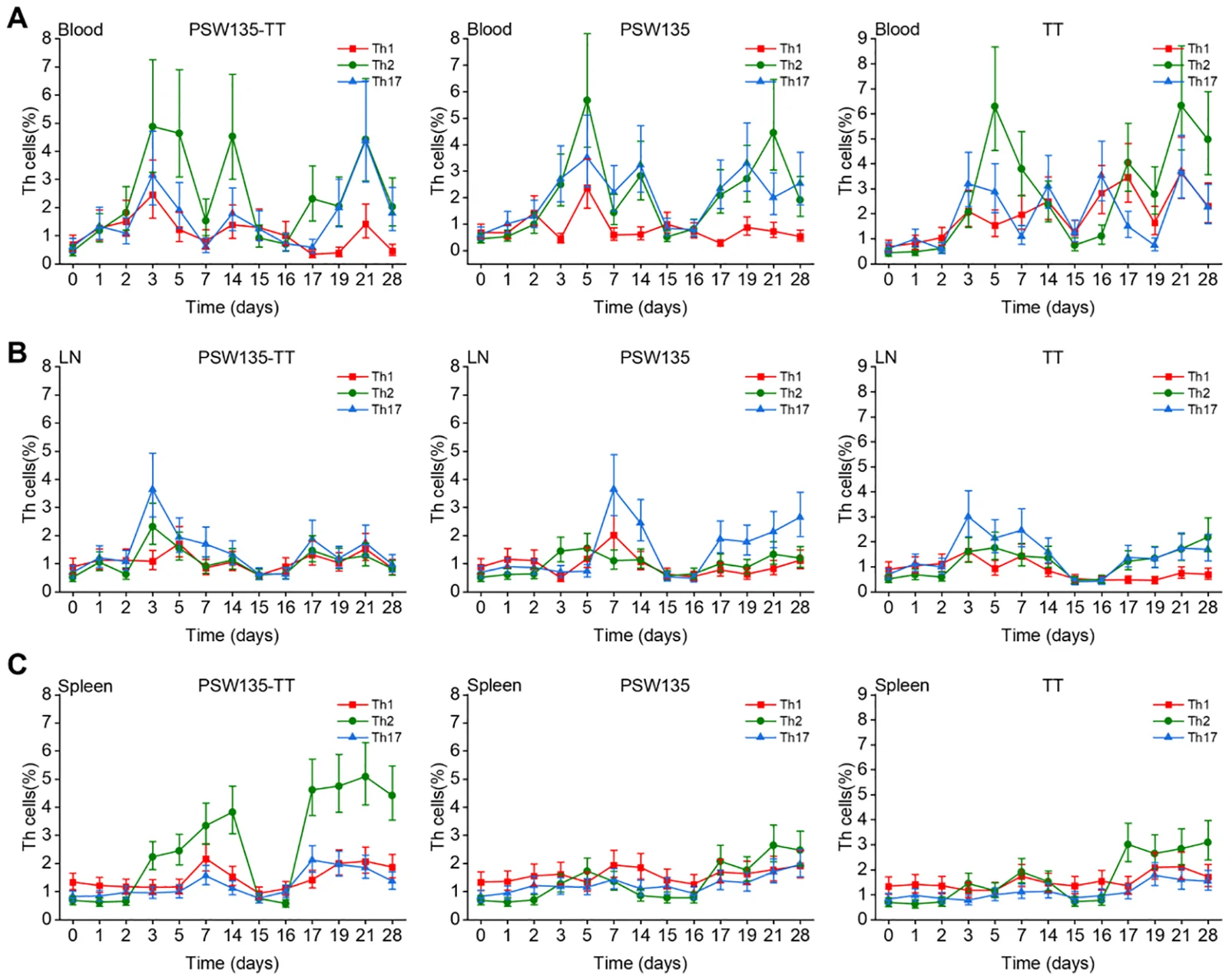
Kinetics of Th1, Th2, and Th17 cell responses induced by non-specific stimulation in blood, lymph nodes (LN), and spleen. Mice (n = 3 per group per time point) received two subcutaneous injections (prime at day 0, boost at day 14) of PSW135-TT, PSW135 alone, or TT alone. Cells harvested from immunized mice at the indicated time points post‑immunization were stimulated with PMA and ionomycin for 4–6 h, followed by intracellular cytokine staining. Frequencies of Th1 (CD4^+^IFN-γ^+^), Th2 (CD4^+^IL-4^+^), and Th17 (CD4^+^IL-17A^+^) cells in **(A)** blood, **(B)** LN, and **(C)** spleen following non-specific stimulation (PMA/ionomycin). Data are presented as estimated marginal means with 95% confidence intervals (error bars). Statistical analysis was performed as described in Section 2.8; full ANOVA results are provided in **Supplementary Table 4A**.

In the blood, Th2 cells in the PSW135-TT group displayed multiple waves: they peaked on day 3 and day 14 after the first immunization, and again on day 21 after the booster immunization (**Figure 5A**). Th17 cells showed two distinct peaks on day 3 and day 21, whereas Th1 cells increased only transiently on day 3 and then declined (**Figure 5A**). In the PSW135-alone group, Th1, Th2, and Th17 cells all peaked simultaneously on day 5 (Th2 > Th17 > Th1); after that, Th2 and Th17 cells decreased and then rose again on day 15, while Th1 cells fluctuated only mildly (**Figure 5A**). Overall, Th2 cells remained consistently higher than Th1 cells. In the TT group, Th2 cells reached a prominent peak on day 5 (higher than Th1 and Th17) and a second peak on day 21; Th1 cells showed a gradual upward trend, whereas Th17 cells fluctuated considerably (**Figure 5A**). The PSW135-TT group induced a multi-wave Th2 response, whereas the Th2 responses induced by PSW135 alone or TT alone were either double-peaked or more concentrated in time.

In the lymph nodes, the kinetics of Th1, Th2, and Th17 cells in the PSW135-TT group were generally similar, with only minor fluctuations; both Th2 and Th17 cells peaked on day 3 (**Figure 5B**). In the PSW135-alone group, Th17 cells displayed a distinct biphasic response, peaking on day 7 and day 16; after day 16, the proportion of Th17 cells exceeded that of Th1 and Th2 cells (**Figure 5B**). In the TT group, Th2 and Th17 cells gradually increased from day 2 onward; Th17 cells remained higher than Th1 and Th2 cells from day 2 to day 14, then increased again thereafter (**Figure 5B**). Overall, Th17 responses in the lymph nodes were more prominent in the PSW135-alone and TT groups, whereas the Th responses in the PSW135-TT group were more balanced.

In the spleen, Th2 cells in the PSW135-TT group peaked on day 14 after the first immunization; after the booster, they rapidly rose again from day 16 and remained at relatively high levels (**Figure 5C**). Th1 and Th17 cells showed a small increase on day 5, then declined, and gradually rose again from day 15 (**Figure 5C**). In the PSW135-alone group, all three Th subsets showed an overall upward trend; after the first immunization, Th1 cells were slightly higher than Th2 cells, but from day 17 after the booster, Th2 cells exceeded Th1 (**Figure 5C**). In the TT group, only Th2 cells increased significantly on day 16 after the booster, reaching levels higher than Th1 and Th17 cells (**Figure 5C**). After the booster immunization, Th2 responses dominated in the spleen, particularly in the PSW135-TT and TT groups.

In summary, all three immunogens induced a Th2-biased immune response, but their kinetic characteristics differed: the PSW135-TT group exhibited multiple Th2 waves in the blood and sustained elevation in the spleen; the PSW135-alone group mainly induced a simultaneous peak of Th1/Th2/Th17 in the blood; and the TT group displayed Th2 predominance in the blood and sustained high levels of Th17 in the lymph nodes.

#### 3.5.2 Th cell subsets induced by PSW135-TT specific stimulation

To evaluate Th cell responses induced by PSW135-TT-specific stimulation, cells isolated from the lymph nodes and spleens of immunized mice were stimulated with PSW135-TT (5 μg/mL) for 72 h. The proportions of Th1 (CD4^+^IFN-γ^+^), Th2 (CD4^+^IL-4^+^), and Th17 (CD4^+^IL-17A^+^) cells were detected by intracellular cytokine staining (n = 3 per group per time point). Two-way ANOVA revealed significant Group × Time interactions for Th1 and Th17 cells in the spleen (**Supplementary Table 4B**). Overall, the proportions of Th cell subsets were higher in the spleen than in the lymph nodes, and Th1 cells outnumbered Th2 cells.

**Figure 6:**
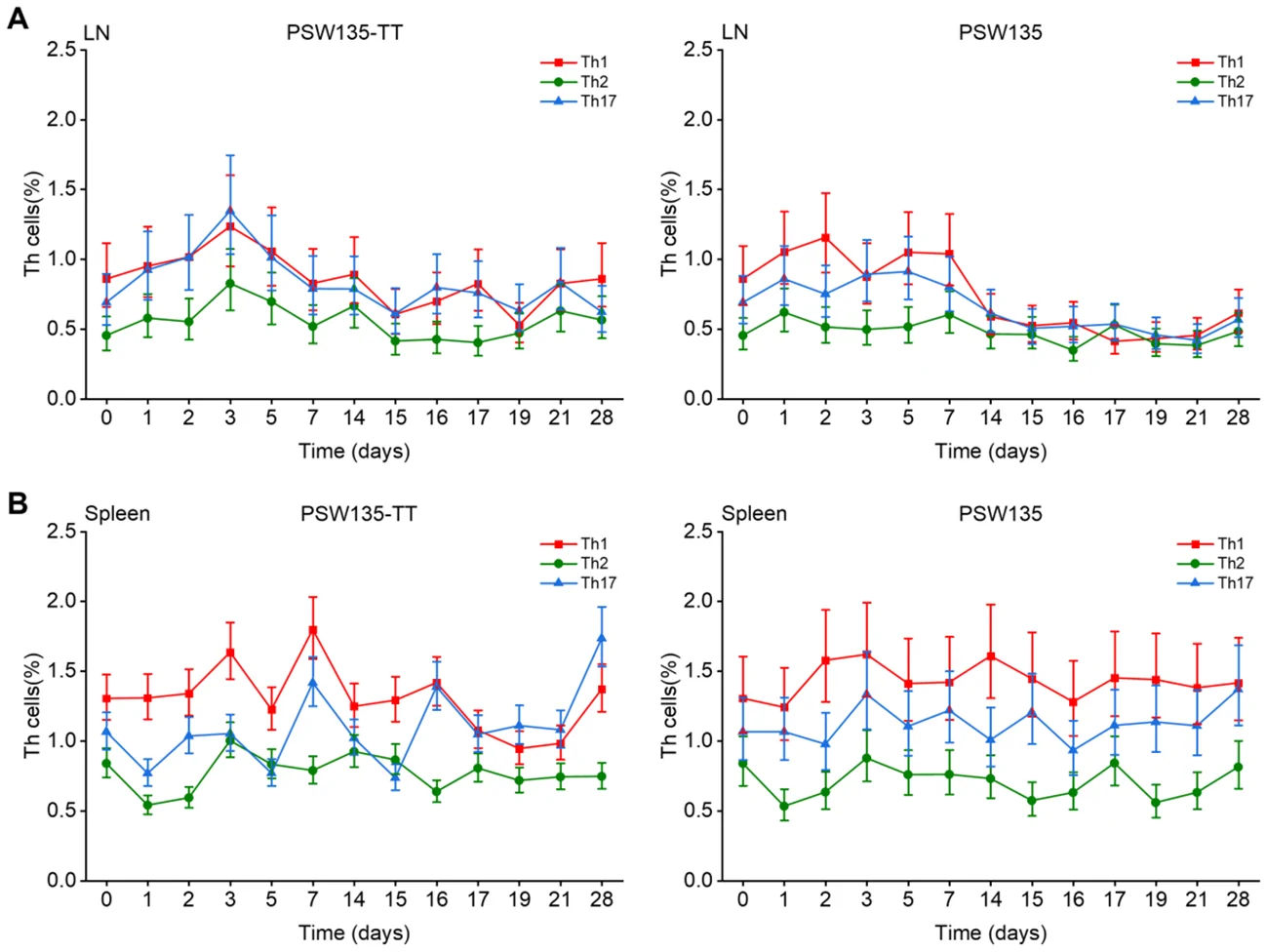
Kinetics of Th1, Th2, and Th17 cell responses induced by PSW135-TT-specific stimulation in lymph nodes (LN) and spleen. Mice (n = 3 per group per time point) received two subcutaneous injections (prime at day 0, boost at day 14) of PSW135-TT, PSW135 alone, or TT alone. Cells harvested from immunized mice at the indicated time points post‑immunization were stimulated with PSW135‑TT (5 mg/mL) for 72 h, followed by intracellular cytokine staining. Frequencies of Th1 (CD4^+^IFN-γ^+^), Th2 (CD4^+^IL-4^+^), and Th17 (CD4^+^IL-17A^+^) cells in **(A)** LN, and **(B)** spleen following PSW135-TT specific stimulation. Data are presented as estimated marginal means with 95% confidence intervals (error bars). Statistical analysis was performed as described in Section 2.8; full ANOVA results are provided in **Supplementary Table 4B**.

In lymph node cells, all three Th subsets fluctuated only mildly in both the PSW135-TT and PSW135 groups, with Th1 and Th17 cells showing slightly higher levels after the first immunization than after the booster, while Th2 cells remained largely unchanged (**Figure 6A**).

In splenocytes, both groups exhibited markedly higher Th1 than Th2 cells. Notably, in the PSW135-TT group, Th17 cells gradually became higher than Th1 cells from day 17 after the booster immunization, whereas in the PSW135 group all three Th subsets displayed stable fluctuations (**Figure 6B**).

#### 3.5.3 Th cell subsets induced by TT specific stimulation

To evaluate Th cell responses induced by TT-specific stimulation, cells isolated from the lymph nodes and spleens of immunized mice were stimulated with TT (2.5 μg/mL) for 72 h. The proportions of Th1 (CD4^+^IFN-γ^+^), Th2 (CD4^+^IL-4^+^), and Th17 (CD4^+^IL-17A^+^) cells were detected by intracellular cytokine staining (n = 3 per group per time point). Two-way ANOVA revealed significant Group × Time interactions for Th1 cells in the spleen (**Supplementary Table 4C**). Overall, Th1 cells outnumbered Th2 cells in both lymph nodes and spleen, and all Th subsets declined from day 0 after immunization, with the most pronounced decline observed for Th1 cells in the lymph nodes of the TT group.

**Figure 7:**
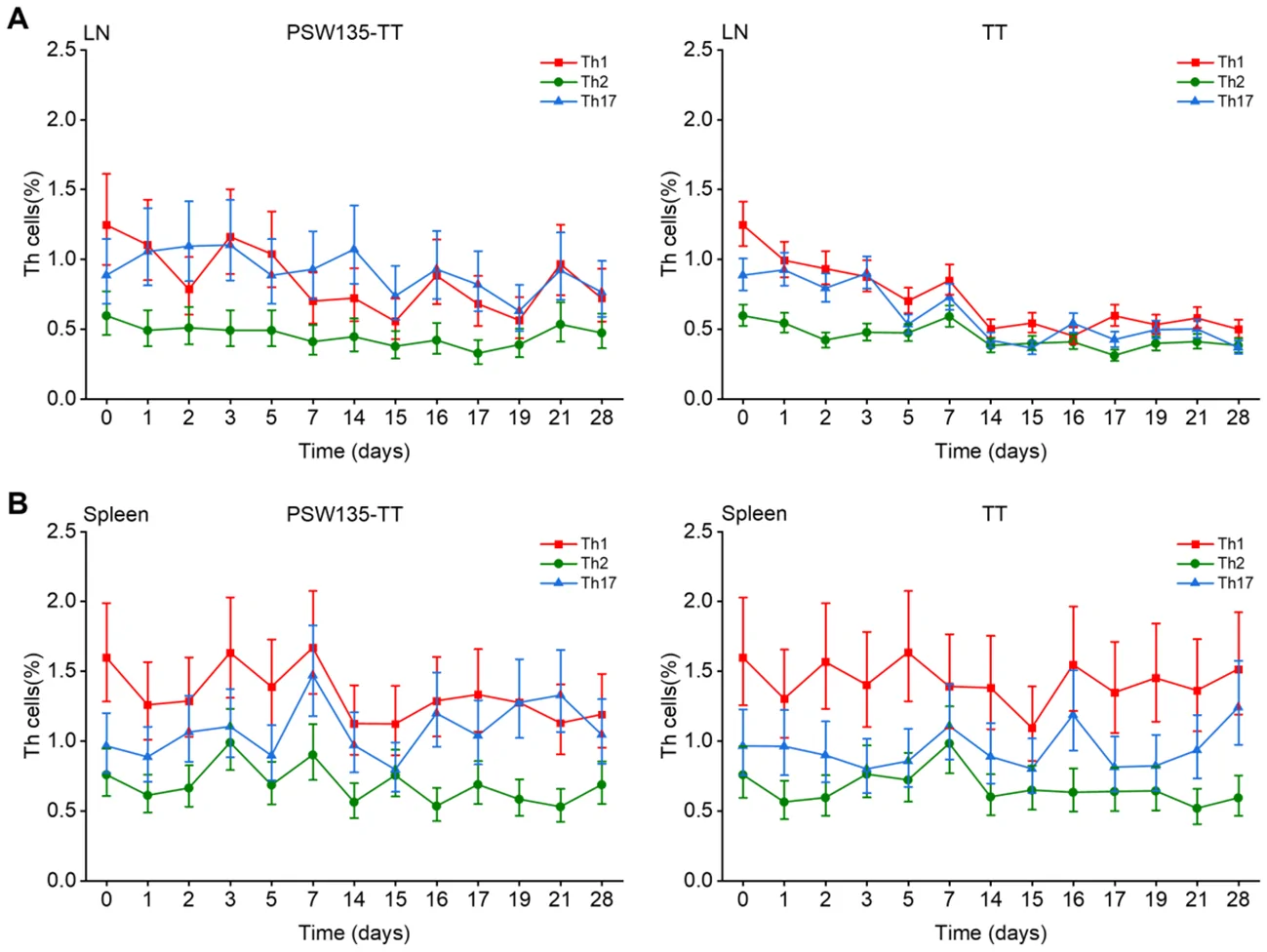
Kinetics of Th1, Th2, and Th17 cell responses induced by TT-specific stimulation in lymph nodes (LN) and spleen. Mice (n = 3 per group per time point) received two subcutaneous injections (prime at day 0, boost at day 14) of PSW135-TT, PSW135 alone, or TT alone. Cells harvested from immunized mice at the indicated time points post‑immunization were stimulated with TT (2.5 mg/mL) for 72 h, followed by intracellular cytokine staining. Frequencies of Th1 (CD4^+^IFN-γ^+^), Th2 (CD4^+^IL-4^+^), and Th17 (CD4^+^IL-17A^+^) cells in **(A)** LN, and **(B)** spleen following TT specific stimulation. Data are presented as estimated marginal means with 95% confidence intervals (error bars). Statistical analysis was performed as described in Section 2.8; full ANOVA results are provided in **Supplementary Table 4C**.

In lymph node cells, Th1 cells showed an overall decreasing trend in both the PSW135-TT and TT groups, and the proportions of Th1 and Th17 cells were roughly comparable (**Figure 7A**).

In splenocytes, Th1 cells in the PSW135-TT group were higher than Th2 and Th17 cells. From day 16 after the booster immunization, Th1 and Th17 cells became roughly equivalent, whereas Th2 cells remained relatively stable (**Figure 7B**).

## 4 Discussion

The present study systematically investigated T-cell and B-cell dynamics following prime-boost immunization with the W135 meningococcal conjugate vaccine (PSW135-TT) in mice. Compared to PSW135 alone or TT alone, the conjugate enhanced humoral and cellular immune responses. Our results show that conjugation of the W135 capsular polysaccharide to tetanus toxoid (TT) enables PSW135 to induce T-cell-dependent antibody responses, and that the immune response was characterized by a predominant IgG1 subclass, consistent with a Th2-biased profile (29).

The antibody kinetics showed that PSW135-TT induced PSW135-specific IgG by day 14, whereas PSW135 alone did not until day 28. The conjugate also maintained higher IgM levels throughout the observation period. All groups exhibited an IgG1 > IgG2a > IgG3 antibody subclass profile, consistent with a Th2-polarized humoral response and with our previous findings (29). These kinetic patterns are consistent with the classical carrier effect described for glycoconjugate vaccines (8, 10), and the earlier appearance of TT-specific IgG (day 7) followed by PSW135-specific IgG (day 14) is compatible with the hapten-carrier sequence. MenACWY-TT vaccination in older adults has been shown to induce functional antibody responses driven by IgM, with increases on days 7 and 14 and a rebound after booster, further supporting our IgM kinetic findings (20).

Analysis of B-cell subsets (CD19^+^ B cells, CD19^+^CD40^+^ cells as a surrogate for B cell activation, and CD19^+^CD138^+^ plasmablast/early plasma cells) in blood, lymph nodes, and spleen revealed that the conjugate induced prolonged B-cell activation across all tissues, whereas PSW135 alone showed a higher late-phase plasmablast/early plasma cell response in the spleen after the booster (reaching the highest splenic level on day 28). The splenic plasmablast/early plasma cell peak on day 7 coincided with the appearance of TT-specific and subsequently PSW135-specific IgG, consistent with the role of T-cell help in antibody production. Recent studies have shown that glycoconjugate vaccines can induce persistent high-affinity antibodies and significantly expand germinal center B cells, mature B cells, and long-lived plasma cells (12, 30). Collectively, these results indicate that conjugation alters the kinetics and sustainability of the B-cell response (25).

T-cell subset analysis showed a consistent CD4^+^ predominance, with higher frequencies in blood and lymph nodes than in the spleen, reflecting normal trafficking patterns (31). The conjugate induced higher T-cell frequencies, most notably in the spleen. The multiple waves of early CD4^+^CD69^+^ activation in the blood (peaking on days 3, 14, and 21 after the first immunization, with a third peak after the booster) may reflect sustained or repeated T-cell stimulation. In the spleen, the delayed CD4^+^CD69^+^ peak in the conjugate group (day 19) compared to TT alone (day 16) may indicate that the polysaccharide moiety may require additional processing and presentation. his possibility is supported by recent studies showing that controlling the speed of antigen transport in dendritic cells can significantly boost vaccine efficacy (32). The triphasic CD4^+^CD25^+^ pattern observed in the conjugate-immunized spleens (peaks on days 3, 19, and 28) suggests prolonged CD4^+^ T-cell activation. The increased CD8^+^CD25^+^ T cells after the booster, particularly in the conjugate group (most pronounced increase from day 17 onward), may reflect CD8^+^ T cell activation; however, the underlying mechanism remains to be determined. Recent work has elucidated the molecular mechanism of cross-presentation, demonstrating that antigens are taken up by dendritic cells, translocated into the cytoplasm, transported to the endoplasmic reticulum, and loaded onto MHC-I molecules (33).

Non-specific stimulation revealed an overall Th2 bias, consistent with the IgG subclass pattern. However, antigen-specific restimulation of splenocytes with the conjugate induced a Th1-dominant recall response. This apparent discrepancy is not contradictory but reflects different immunological dimensions: non-specific stimulation measures the global polarization of the immune environment, whereas antigen-specific restimulation directly measures the functional capacity of antigen-experienced T cells. Vaccine-induced T cells in tissues are highly heterogeneous, possessing both effector and regulatory functional programs, including Th1/Th2/Th17 cytokine production (31). Notably, the conjugate induced a late Th17 increase after the booster, which may be associated with mucosal immunity and protection against encapsulated bacteria, as suggested by previous studies (27, 28). TT-specific stimulation showed Th1 > Th2 in both lymph node and spleen cells, with PSW135-TT showing higher Th1 responses than TT alone.

Collectively, these findings are consistent with a stepwise model of conjugate vaccine-induced immunity. Upon immunization, the conjugate may be taken up by antigen-presenting cells, potentially leading to CD4^+^ T cell activation (as suggested by multiple CD69^+^ waves) and Th2 polarization. These activated TT-specific T cells may then provide help to PSW135-specific B cells, contributing to B-cell activation and differentiation into plasmablasts/early plasma cells. After the booster, a triphasic CD4^+^CD25^+^ activation pattern was observed, accompanied by Th1- and Th17-skewed T cell responses and enhanced antibody production. This model of stepwise T-B cell collaboration is consistent with the observation that conjugation to a carrier protein generates a robust antibody response and a mixed T-cell compartment, as previously described (31).

Together, these results extend our previous findings by providing a comprehensive kinetic analysis of B-cell activation, T-cell dynamics, and Th subset polarization (29). Several limitations should be acknowledged. First, the study lacked a control group receiving unconjugated PSW135 mixed with TT, which would have helped distinguish conjugation-specific effects from simple co-administration of both antigens. Second, the PSW135-alone group received a higher dose of polysaccharide (12.5 μg) compared to the amount present in the conjugate formulation (1.25 μg). This dose was chosen based on preliminary experiments, as unconjugated PSW135 alone at lower doses failed to elicit detectable antibody responses; however, this dose discrepancy should be considered when interpreting group comparisons. Third, we did not perform serum bactericidal assays at each time point, though our previous work confirmed bactericidal activity. Fourth, we did not assess Tfh or germinal center B cells, nor did we perform multiplex cytokine quantification. Fifth, the sample size for cellular analyses was limited to three mice per group per time point, which may affect the generalizability of the findings. Nevertheless, the large effect sizes (η²p > 0.14) observed for most significant comparisons support the robustness of the results. Sixth, in the antigen-specific restimulation experiments (**Figures 6**, **7**), we did not include PSW135-only, TT-only, unstimulated, or irrelevant-antigen controls. Therefore, we have limited our interpretation to “conjugate-induced T cell recall responses” and acknowledge that we cannot definitively distinguish polysaccharide-specific from carrier-specific T cell responses. Seventh, the study used naïve mice, whereas humans may have pre-existing TT immunity from routine DTP vaccination, which could affect the carrier effect (34). Future studies should address these points and test protective efficacy against live bacterial challenge.

In summary, prime-boost immunization with PSW135-TT was associated with accelerated IgG class switching, sustained B-cell activation, repeated CD4^+^ and CD8^+^ T-cell activation, Th1-skewed T cell responses with a late Th17 boost, and a Th2-biased antibody response. This study provides comprehensive immunological evidence supporting PSW135-TT as a vaccine candidate against W135 meningococcal disease.

## Data Availability

The raw data supporting the conclusions of this article will be made available by the authors, without undue reservation.
