# Supplementary material for: T-cell and B-cell dynamics following prime-boost immunization with W135 meningococcal conjugate vaccine in mice
Source: Front Immunol. 2026 Aug 19;17:1900097. doi: 10.3389/fimmu.2026.1900097 (PMC13534131; doi:10.3389/fimmu.2026.1900097)
Supplement: Supplementary file 1 [file DataSheet1.docx]

Supplementary Figure S1. Representative gating strategy for flow cytometric analysis.

Representative flow cytometry gating strategy for lymphocytes isolated from blood, draining inguinal lymph nodes (LN), and spleen. Representative flow cytometry gating strategy for lymphocytes isolated from blood, draining inguinal lymph nodes (LN), and spleen. Lymphocytes were gated on forward scatter area versus side scatter area (FSC‑A vs SSC‑A). From the lymphocyte gate, CD3⁺ T cells and CD19⁺ B cells were identified. CD4⁺ and CD8⁺ subsets were further gated from CD3⁺ T cells, and activation markers CD69⁺ and CD25⁺ were analyzed on CD4⁺ and CD8⁺ T cells. Plasmablast/early plasma cells were identified as CD19⁺CD138⁺ from the CD19⁺ B cell gate. All gates were set based on fluorescence‑minus‑one (FMO) controls.
